# Machine learning-based prediction of early neurological deterioration after intravenous thrombolysis for stroke: insights from a large multicenter study

**DOI:** 10.3389/fneur.2024.1408457

**Published:** 2024-09-09

**Authors:** Rui Wen, Miaoran Wang, Wei Bian, Haoyue Zhu, Ying Xiao, Jing Zeng, Qian He, Yu Wang, Xiaoqing Liu, Yangdi Shi, Linzhi Zhang, Zhe Hong, Bing Xu

**Affiliations:** ^1^Second Affiliated Hospital of Chongqing Medical University, Chongqing, China; ^2^Affiliated Central Hospital of Shenyang Medical College, Shenyang Medical College, Shenyang, China; ^3^Shenyang First People’s Hospital, Shenyang Medical College, Shenyang, China; ^4^Shenyang Tenth People’s Hospital, Shenyang Medical College, Shenyang, China; ^5^Chongqing Medical University, Chongqing, China

**Keywords:** machine learning, early neurological deterioration (END), intravenous thrombolysis (IVT), multicenter study design, prediction model

## Abstract

**Background:**

This investigation seeks to ascertain the efficacy of various machine learning models in forecasting early neurological deterioration (END) following thrombolysis in patients with acute ischemic stroke (AIS).

**Methods:**

Employing data from the Shenyang Stroke Emergency Map database, this multicenter study compiled information on 7,570 AIS patients from 29 comprehensive hospitals who received thrombolytic therapy between January 2019 and December 2021. An independent testing cohort was constituted from 2,046 patients at the First People’s Hospital of Shenyang. The dataset incorporated 15 pertinent clinical and therapeutic variables. The principal outcome assessed was the occurrence of END post-thrombolysis. Model development was executed using an 80/20 split for training and internal validation, employing classifiers like logistic regression with lasso regularization (lasso regression), support vector machine (SVM), random forest (RF), gradient-boosted decision tree (GBDT), and multi-layer perceptron (MLP). The model with the highest area under the curve (AUC) was utilized to delineate feature significance.

**Results:**

Baseline characteristics showed variability in END incidence between the training (*n* = 7,570; END incidence 22%) and external validation cohorts (*n* = 2,046; END incidence 10%; *p* < 0.001). Notably, all machine learning models demonstrated superior AUC values compared to the reference model, indicating their enhanced predictive capacity. The lasso regression model achieved the highest AUC at 0.829 (95% CI: 0.799–0.86; *p* < 0.001), closely followed by the MLP model with an AUC of 0.828 (95% CI: 0.799–0.858; *p* < 0.001). The SVM, RF, and GBDT models also showed commendable AUCs of 0.753, 0.797, and 0.774, respectively. Decision curve analysis revealed that the SVM and MLP models demonstrated a high net benefit. Feature importance analysis emphasized “Onset To Needle Time” and “Admission NIHSS Score” as significant predictors.

**Conclusion:**

Our research establishes the MLP and lasso regression as robust tools for predicting early neurological deterioration in acute ischemic stroke patients following thrombolysis. Their superior predictive accuracy, compared to traditional models, highlights the significant potential of machine learning approaches in refining prognosis and enhancing clinical decisions in stroke care management. This advancement paves the way for more tailored therapeutic strategies, ultimately aiming to improve patient outcomes in clinical practice.

## Introduction

Early neurological deterioration (END) is a common and critical outcome in patients following an ischemic stroke, marked by a rapid decline in neurological function within the first few days after stroke onset. Identifying individuals at heightened risk for END carries significant clinical implications, from aiding clinicians in informed therapeutic decision-making, providing accurate prognostic information to patients and their families, to tailoring surveillance and intervention strategies.

Despite the critical impact of END, the predictive tools currently available for assessing its occurrence, especially after interventions like intravenous thrombolysis (IVT) or endovascular treatment (EVT), are limited and often lack validation in diverse patient populations (1–4). To ensure these models’ integration into clinical workflows, they must undergo rigorous evaluation, including external validation to establish their wide applicability. While certain biomarkers and clinical indicators have been identified as associated with END risk post-thrombolysis (2, 5–7), the generalizability of these models remains constrained by their limited validation across different demographic and clinical contexts. This challenge underscores the necessity for research to bridge this gap through developing and validating models on broad, multi-center data that can more accurately predict END across various patient cohorts.

The advent of machine learning offers a promising avenue to address these limitations by surpassing traditional analytical methods in risk assessment (8–10). Leveraging computational algorithms to sift through large, complex datasets with numerous, multi-dimensional variables, machine learning can uncover intricate, non-linear relationships between clinical characteristics, thus enhancing the accuracy of prognostic predictions (11). Despite machine learning techniques demonstrating superior predictive capabilities over conventional statistical models in various medical domains, their application in predicting END following ischemic stroke, particularly with large-scale, multicenter datasets, remains underexplored.

In response to these challenges, our study aims to fill these gaps by analyzing data collected from multiple centers across different regions to develop machine learning-based models for predicting the likelihood of END after intravenous thrombolysis. We hypothesize that our models will offer improved accuracy and applicability across varied patient demographics compared to existing risk assessment tools. Further, we plan to externally validate our models’ predictive power in a real-world clinical setting, targeting patients treated with intravenous thrombolysis. Our research seeks to advance patient management strategies by enhancing our ability to predict and thereby mitigate the risk of END in the aftermath of an ischemic stroke.

## Methods

This research is structured as a retrospective, multi-center, observational cohort study, utilizing data collected from various major hospitals. Its core objective is to assess and contrast the performance of diverse machine learning models in forecasting the outcomes of patients who have undergone thrombolysis treatment for ischemic stroke. Adhering to ethical standards and preserving the integrity of the research, the study has been granted approval by the Research Ethics Committee of Shenyang First Hospital, with the approval number being 2023SYKYPZ52.

### Datasets

Our study commenced by utilizing the Shenyang Stroke Emergency Map database, an expansive resource serving over 9 million people, pivotal to the city’s efforts in elevating stroke care standards. Data from 30 comprehensive hospitals were meticulously recorded into this database by specialized staff. For our analysis, we concentrated on a curated group of 8,924 acute ischemic stroke (AIS) patients who received thrombolytic treatment, selecting data from 29 of these hospitals. This segment of our research covered the period from January 2019 to December 2021, providing a rich dataset for developing predictive models.

Firstly, we excluded patients who had undergone endovascular treatment, as recorded in the database. Further, patients missing data on either their admission NIHSS score or post-thrombolysis NIHSS score were removed. Beyond these specific data-related exclusion criteria, we also excluded patients with severe organ dysfunctions, such as heart, liver, and kidney issues, as well as those with malignant tumors or other significant infections. Additionally, cases with other missing key feature data or those with poor data quality were also disregarded.

Subsequently, an additional independent testing cohort was established, comprising 2,173 consecutive patients who received thrombolytic therapy at the First People’s Hospital of Shenyang during the identical timeframe.

Following the stringent patient selection criteria, our data preparation encountered an anticipated challenge: missing values. Within the scope of our study, absent data for both predictor and outcome variables were evident. Such omissions may be attributed to various reasons, such as unperformed tests or incomplete patient records. To address this intricacy, we adopted the multiple imputation by chained equations (MICE) approach, employing the mice package in R (12). During the imputation process, we incorporated all predictor variables and the outcome variable, opting for the random forest (RF) method owing to its adeptness in deciphering intricate data patterns. It is noteworthy that, despite the inherent complexity of our dataset, we abstained from introducing interaction terms in the imputation model.

We proceeded with the creation of 20 imputed datasets. For the purpose of aggregation, the imputed values across these datasets were averaged, with categorical variables determined by mode. Post-imputation, a thorough analysis of each dataset ensued. To consolidate the findings, Rubin’s rules (13) were meticulously applied, yielding a harmonized output. To corroborate the robustness of our imputation technique, a sensitivity analysis juxtaposing the imputed and the original data sets was performed, the details of which have been annexed.

To further ensure the robustness of our imputation technique, we conducted a sensitivity analysis by comparing the imputed datasets with the original data, which demonstrated the reliability of the imputation process. Detailed results and visualizations of this comparison, including distribution analyses, odds ratio comparisons, and the data imputation workflow, have been included in the Supplementary material for comprehensive reference.

### Predictors

The structured dataset encapsulated 15 variables. This included 12 clinical metrics: gender, age, post awakening stroke, in hospital stroke, body mass index (BMI), systolic blood pressure (SBP), diastolic blood pressure (DBP), admission mRS score, admission NIHSS score, swallowing function score, onset to needle time (ONT), and TOAST classification. Additionally, there were three therapeutic metrics: thrombolytic drug, antiplatelet therapy, and anticoagulation therapy.

### The primary outcomes

The primary outcome was the occurrence of END following thrombolysis. END was defined as a neurological deterioration with a ≥ 4 points increase on the NIHSS score compared with baseline within 24 h after the treatment (14).

### Model development and validation

The training cohort was arbitrarily divided into two subsets: 80% for model training and 20% for internal validation. In this phase, five machine learning classifiers were employed: logistic regression with lasso regularization (lasso regression), support vector machine (SVM), random forest (RF), gradient-boosted decision tree (GBDT), and multi-layer perceptron (MLP). The reference model used a logistic regression with no regularization.

The classifiers were selected based on their established performance in binary classification tasks, particularly within clinical settings. Lasso regression was chosen for its simplicity and interpretability, allowing for straightforward identification of significant predictors. The SVM was included due to its effectiveness in handling high-dimensional data and its robustness in classification tasks. RF and GBDT are both ensemble methods known for their ability to manage complex interactions and their strong performance across various datasets. The MLP, a neural network-based model, was selected for its capability to capture intricate nonlinear relationships between features. Each of these models was expected to contribute uniquely to the prediction of early neurological deterioration in stroke patients following thrombolysis.

Prior to model training, we standardized all continuous variables using the StandardScaler from scikit-learn to ensure that each feature contributed equally to the analysis, thus enhancing model performance. This preprocessing step was meticulously documented and performed separately for the training and testing datasets to prevent data leakage.

An exhaustive grid search, coupled with cross-validation, was conducted to optimize the hyperparameters for these classifiers within predefined ranges. This rigorous approach helped us to determine the most effective model parameters based on the highest area under the receiver operating characteristic curve (AUC) achieved on the internal validation set.

In the training cohort, which consisted of 80% randomly selected samples, we constructed a reference model and the five aforementioned proprietary machine learning models for each outcome. Each model’s performance was rigorously evaluated in the external validation cohort using a spectrum of metrics, including the mean AUC and decision curve analysis, to identify the best-performing model for each study outcome.

The decision curve analysis was particularly crucial as it provided a quantifiable measure of the trade-offs between the risks of undertriage (false negatives) and overtriage (false positives), given a threshold probability or clinical preference (15, 16). This analysis facilitated the calculation of the net benefit for each model across a range of threshold probabilities for the outcome, which was graphically represented in the decision curves included in our results.

### Feature importance

The model exhibiting the highest AUC was chosen to highlight the importance of features, ensuring insights into the most influential predictors.

### Statistical analysis

Categorical variables are delineated as count (%) and continuous variables as mean (SD) or median (IQR). The presence of a normal distribution was confirmed by the Kolmogorov–Smirnov test. We employed the t-test to evaluate disparities between parametric continuous variables, the Mann–Whitney *U* test for non-parametric variables, the *χ*^2^ test for categorical variables, and the Fisher exact test for 2 × 2 tables. No correction for multiple testing was instituted. A two-sided *p* < 0.05 was deemed statistically significant. All analyses were conducted with R version 4.1.2 and Python version 3.10.2.

## Results

Table 1 illustrates the baseline characteristics of patients from the training cohort (*n* = 7,220) and the external validation cohort (*n* = 1,921). The training cohort initially had 8,924 samples, with 1,534 excluded for endovascular treatment, 73 for missing admission NIHSS scores, and 97 for not receiving thrombolysis. The external validation cohort began with 2,173 patients, excluding 249 for endovascular treatment, 1 for a missing NIHSS score, and 2 for not receiving thrombolysis. Both groups showed a similar median age of 65 years and a gender distribution where females accounted for approximately 30%. Notably, there was a significant difference in in-hospital stroke rates, with the training cohort having 5.2% compared to 1.5% in the external validation cohort. Variations were also observed in clinical metrics like SBP, DBP, swallowing function score, and TOAST classification. The use of recombinant tissue plasminogen activator (rt-PA) was predominant in the training cohort (88%), while urokinase was more frequently used in the validation cohort (35%). The END incidence was slightly higher in the training cohort at 22% compared to the validation cohort’s 10%. Overall, while some demographic characteristics aligned between the cohorts, clinically relevant variations highlight the significance of external validation in model assessments.

**Table 1:** Baseline features of included cohorts.

Characteristic	Training cohort (*N* = 7,220)	External validation cohort (*N* = 1,921)	*p*-value
Gender			0.9
Male	5,062 (70%)	1,351 (70%)	
Female	2,155 (30%)	570 (30%)	
Unknown	3	0	
Age, median (Q1, Q3), years	65 (57, 72)	65 (58, 72)	0.3
Unknown	3	0	
Post awakening stroke			0.14
No	7,031 (97%)	1,882 (98%)	
Yes	189 (2.6%)	39 (2.0%)	
In hospital stroke			<0.001
No	6,831 (95%)	1,892 (98%)	
Yes	389 (5.4%)	29 (1.5%)	
BMI	24.2 (21.9, 26.2)	24.1 (22.1, 26.1)	0.7
Unknown	527	37	
SBP, (Q1, Q3), mmhg	157 (141, 169)	149 (138, 160)	<0.001
DBP, (Q1, Q3), mmhg	90 (80, 97)	85 (79, 93)	<0.001
Admission mRS score			
0	3,271 (53%)	546 (28%)	
1	845 (14%)	386 (20%)	
2	487 (7.9%)	535 (28%)	
3	416 (6.7%)	195 (10%)	
4	925 (15%)	191 (9.9%)	
5	216 (3.5%)	67 (3.5%)	
6	15 (0.2%)	1 (<0.1%)	
Unknown	1,045	0	
Admission NIHSS score	6 (4, 11)	3 (2, 6)	<0.001
Swallowing function score			<0.001
1	2,286 (45%)	1,131 (93%)	
2	1,706 (34%)	82 (6.7%)	
3	387 (7.7%)	1 (<0.1%)	
4	351 (7.0%)	1 (<0.1%)	
5	314 (6.2%)	1 (<0.1%)	
Unknown	2,176	705	
TOAST classification			<0.001
LAA	3,768 (52%)	1,519 (79%)	
CE	607 (8.4%)	94 (4.9%)	
SAA	2,329 (32%)	282 (15%)	
SOE	35 (0.5%)	3 (0.2%)	
SUE	481 (6.7%)	23 (1.2%)	
Thrombolytic drug			<0.001
rt-PA	6,384 (88%)	1,211 (63%)	
Urokinase	746 (10%)	663 (35%)	
Others	90 (1.2%)	47 (2.4%)	
ONT, median (Q1, Q3), minutes	170 (125, 225)	158 (113, 222)	<0.001
Unknown	49	0	
Antiplatelet therapy			<0.001
No	797 (11%)	112 (5.8%)	
Yes	6,177 (89%)	1,809 (94%)	
Unknown	246	0	
Anticoagulation therapy			<0.001
No	6,506 (94%)	1,890 (99%)	
Yes	388 (5.6%)	28 (1.5%)	
Unknown	326	3	
END, *n* (%)	1,574 (22%)	192 (10.0%)	<0.001

BMI, body mass index; SBP, systolic blood pressure; DBP, diastolic blood pressure; LAA, large-artery atherosclerosis stroke; CE, cardioembolic stroke; SAA, small artery occlusion or lacunar stroke; SOE, stroke of other determined etiology; SUE, stroke of undetermined etiology; ONT, onset-to-needle time; END, early neurological deterioration; rt-PA, recombinant tissue plasminogen activator.

**Table 2:** Predictive ability of the reference model and 5 machine learning models for END in patients with ischemic stroke who received thrombolysis.

Model	AUC with CI	*p*-value	Sensitivity (95% CI)	Specificity (95% CI)	PPV (95% CI)	NPV (95% CI)	PLR (95% CI)	NLR (95% CI)
Reference model	0.614 (0.564–0.664)	[Reference]	0.602 (0.556–0.657)	0.835 (0.737–0.925)	0.21 (0.161–0.338)	0.927 (0.92–0.939)	2.388 (1.732–4.6)	0.707 (0.582–0.785)
Logistic regression with lasso regularization	0.829 (0.799–0.86)	<0.001	0.775 (0.708–0.828)	0.775 (0.719–0.825)	0.550 (0.511–0.587)	0.775 (0.729–0.811)	0.368 (0.332–0.402)	0.307 (0.259–0.353)
Random forest	0.797 (0.764–0.829)	<0.001	0.759 (0.640–0.870)	0.702 (0.586–0.809)	0.461 (0.408–0.511)	0.744 (0.663–0.800)	0.294 (0.252–0.337)	0.224 (0.166–0.290)
GBDT	0.774 (0.741–0.808)	<0.001	0.728 (0.604–0.849)	0.697 (0.575–0.806)	0.425 (0.366–0.485)	0.718 (0.631–0.784)	0.271 (0.227–0.315)	0.207 (0.151–0.265)
MLP	0.828 (0.799–0.858)	<0.001	0.773 (0.672–0.865)	0.734 (0.641–0.824)	0.507 (0.454–0.553)	0.763 (0.695–0.814)	0.329 (0.287–0.376)	0.261 (0.203–0.332)
SVM	0.753 (0.711–0.795)	<0.001	0.676 (0.562–0.802)	0.755 (0.608–0.848)	0.431 (0.366–0.492)	0.687 (0.601–0.760)	0.290 (0.232–0.347)	0.240 (0.155–0.314)

**Figure 1:**
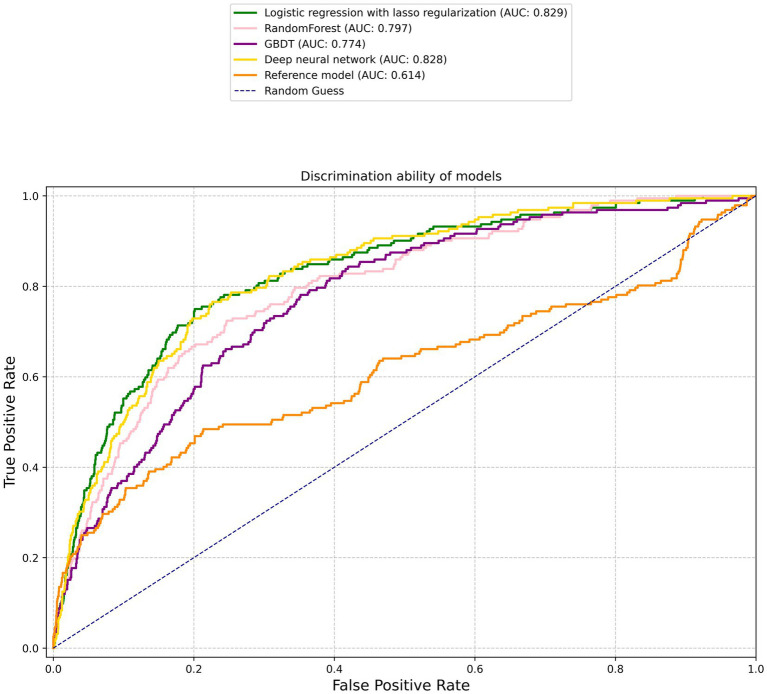
Receiver operating characteristic curves. The corresponding values of the area under the curve for each model are presented in Table 2.

Figure 1 illustrates the discriminative abilities of the various models through the depiction of their ROC curves. The reference model showed limited efficacy, with a C statistic of 0.614 (95% CI, 0.564–0.664). Contrary to expectations, the performance of machine learning models significantly surpassed that of the reference. Specifically, the lasso regression model and MLP model demonstrated superior performances, both achieving a C statistic of 0.829 (95% CI, 0.799–0.86) and 0.828 (95% CI, 0.799–0.858) respectively, with *p*-values less than 0.001. The RF model also performed well, with a C statistic of 0.797 (95% CI, 0.764–0.829), and the GBDT model followed closely with a C statistic of 0.774 (95% CI, 0.741–0.808). The SVM model, while slightly lagging, still displayed commendable predictive ability with a C statistic of 0.753 (95% CI, 0.711–0.795). All machine learning models showed statistically significant improvements over the reference model, underscoring the potential of advanced analytical techniques in enhancing ischemic stroke outcome predictions.

**Figure 2:**
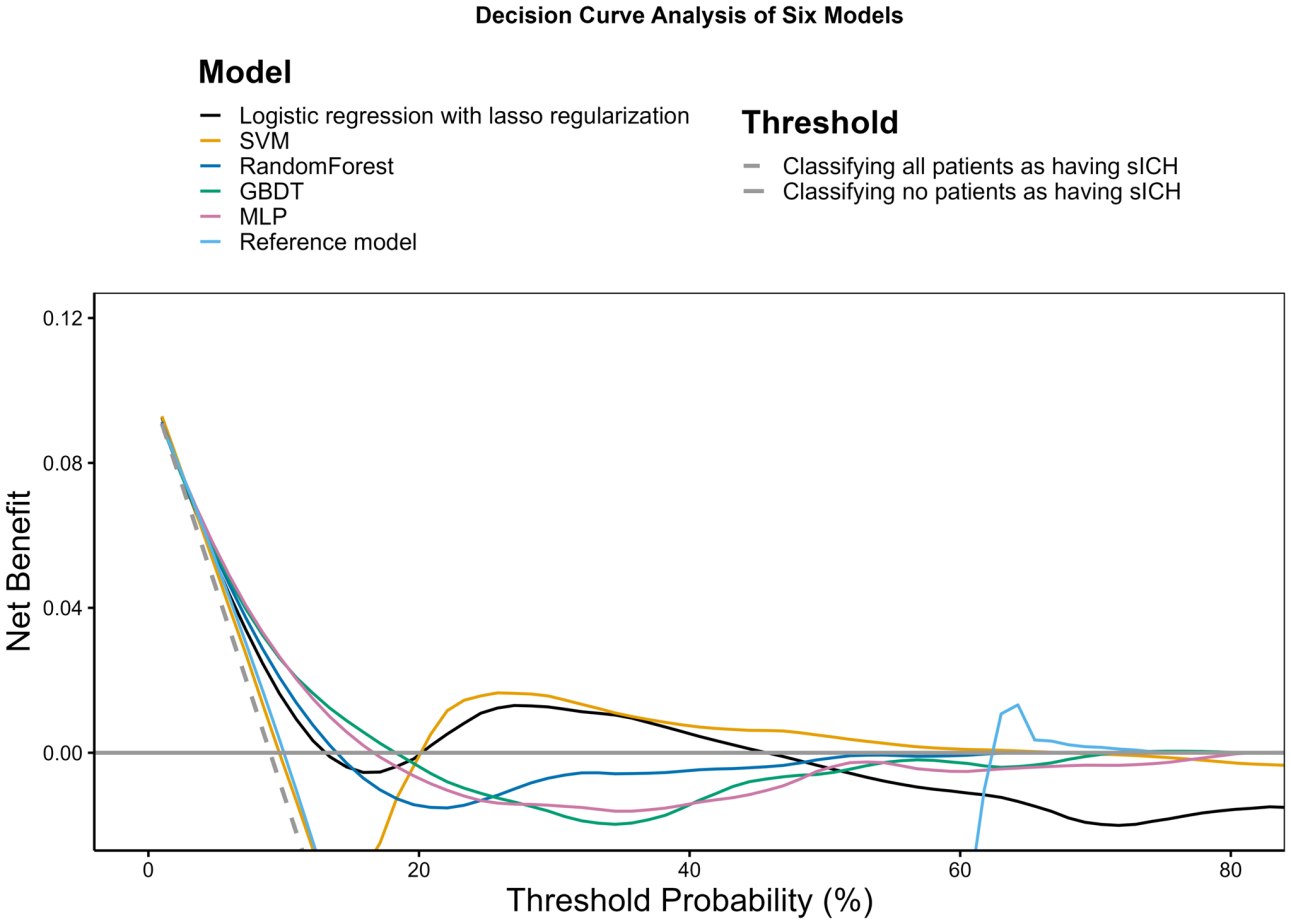
Decision curve analysis. The *x*-axis indicates the threshold probability for hospitalization outcome. The *y*-axis indicates the net benefit. The curves (decision curves) indicate the net benefit of models (the reference model and 5 machine learning models) as well as 2 clinical alternatives (classifying no patients as having END vs. classifying all patients as having END) over a specified range of threshold probabilities of outcome. Only the SVM and MLP models exhibited a positive net benefit.

Based on the decision curve analysis depicted in Figure 2, it is observed that the lasso regression, RF, GBDT, MLP, and SVM models demonstrate a net benefit over the range of threshold probabilities. Among these, the MLP model shows a notably extensive range of threshold probabilities where it offers net benefits, surpassing the other models in its utility. The RF and GBDT models also provide net benefits, although to a lesser extent compared to the MLP model. The reference model, in contrast, appears to have no net benefit across the examined threshold probabilities, suggesting that the machine learning approaches, especially the MLP model, may be more beneficial in clinical decision-making contexts.

**Figure 3:**
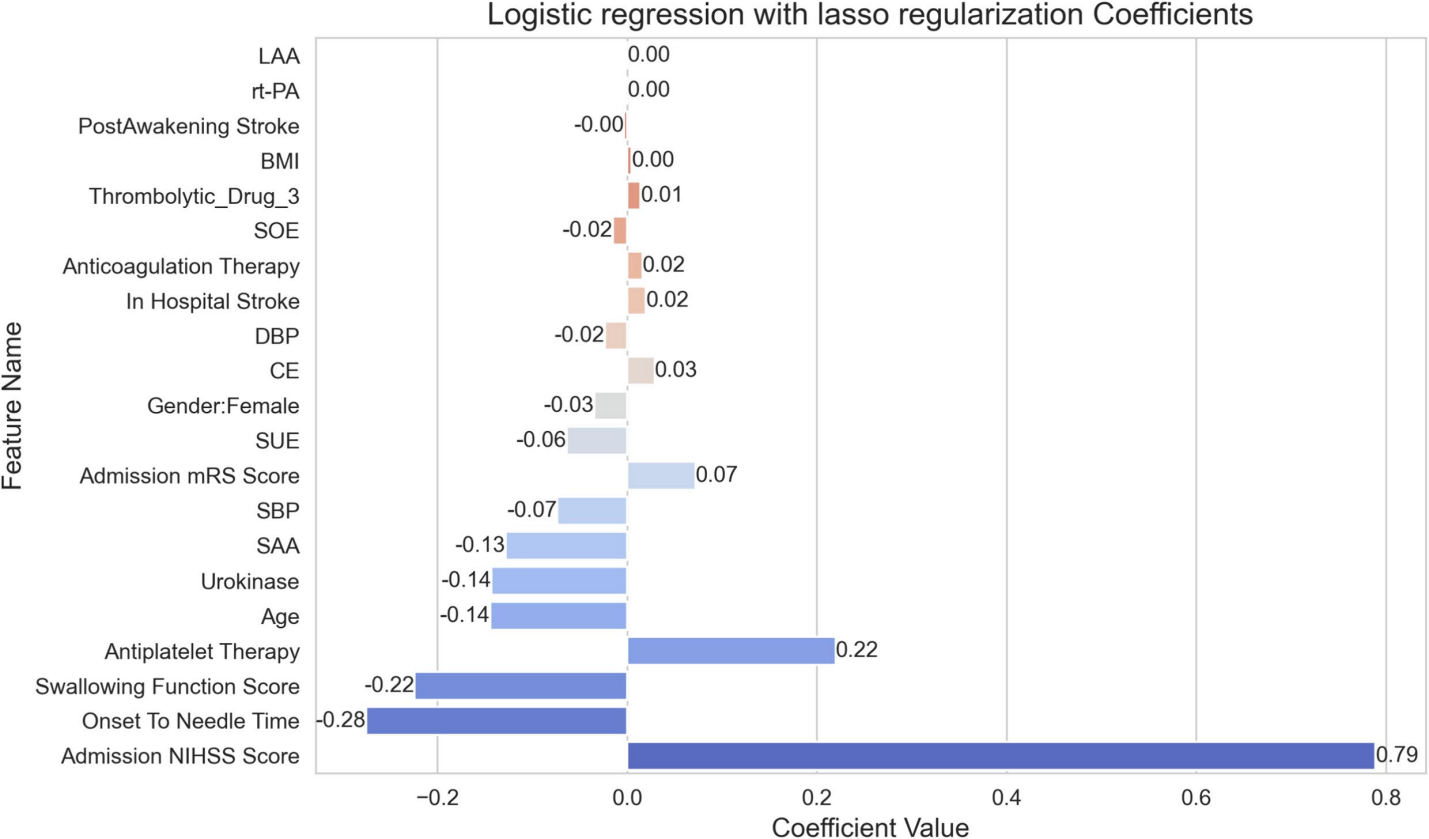
Importance of features as determined by logistic regression with lasso regularization.

In Figure 3, lasso regression model’s coefficients reveals the relative importance of each feature in determining the outcome. This model, selected for its superior AUC value, highlights the discriminative strength of the features involved in the prediction. The variable indicating “Onset To Needle Time” shows the most significant negative impact on the outcome with a coefficient of −0.28, implying a reduced likelihood of a favorable outcome as the time to treatment increases. In contrast, the “Admission NIHSS Score” is identified as the most influential positive factor with a coefficient of 0.79, suggesting that higher scores on admission are strongly predictive of the outcome under study. Other notable features influencing the outcome positively are the “Swallowing Function Score” and “Antiplatelet Therapy” with coefficients of −0.22 and −0.14 respectively, which, despite being negative, indicate less impact than the time to treatment. Noteworthy detractors from a favorable outcome include “Age” and “Urokinase,” each having a coefficient of −0.14, indicating that these factors contribute to a decrease in the likelihood of a positive outcome. Conversely, “Gender: Female” and “DBP” show less substantial influence on the prediction, each bearing a slightly negative coefficient value. These smaller coefficients suggest a more nuanced effect on the outcome.

This model thus provides a quantitative analysis of how various clinical factors may impact the probability of patient outcomes following treatment, offering clinicians a data-driven means of assessing patient prognosis.

## Discussion

In our study, the analysis of predictive models for END following thrombolysis revealed the lasso regression and MLP models as standout performers, with high AUC values indicative of their robust discriminative abilities. These findings underscore the potential utility of machine learning algorithms in clinical settings, providing an advanced tool for clinicians in the prognosis of ischemic stroke outcomes. Particularly, the robustness of the lasso regression suggests that a more simplified, interpretable approach does not compromise the predictive quality. This not only aligns with contemporary efforts to leverage big data in improving patient care but also offers a feasible pathway for integrating complex statistical models into everyday clinical decision-making, thereby enhancing the precision of post-thrombolysis END risk stratification.

Comparative analysis with previous studies situates our findings within the broader context of ongoing research into END post-thrombolysis. Our machine learning approach, particularly the lasso regression, has shown predictive superiority with its high AUC value, which aligns with earlier studies that established a variety of clinical and biological markers as significant predictors of END (1, 2, 5). However, unlike these studies, which often focused on singular predictive factors such as the neutrophil to lymphocyte ratio (NLR) (5), our model integrates multiple variables, enhancing predictive accuracy. Notably, previous research has yielded predictive tools such as simple scores or nomograms with considerable success (1, 17). Our study extends this by utilizing advanced machine learning techniques, which may offer an improvement over traditional statistical methods. The significant contribution of admission NIHSS scores to outcome prediction in our model is consistent with earlier findings (2). Yet, in contrast to some previous models which did not achieve widespread clinical adoption possibly due to complexity or lack of validation (4), our model combines accuracy with practical applicability. Our findings advocate for a more nuanced understanding of END, corroborating the multifactorial nature of this adverse event and underscoring the need for comprehensive tools that accommodate the complexity of stroke outcomes (18).

The clinical relevance of our study is particularly pronounced in the arena of post-thrombolysis care for ischemic stroke patients. The high accuracy of the lasso regression and MLP models in predicting END signifies a crucial advancement in stroke management. By employing these models, clinicians can potentially determine the likelihood of END more precisely, enabling more personalized care. For patients at high risk, this could mean intensified monitoring, timely interventions, and informed decision-making regarding secondary prevention strategies. Conversely, for those deemed at lower risk, it might help in averting unnecessary treatments and the associated costs and side effects. Furthermore, the models’ capacity to process complex, multidimensional data reflects the real-world clinical scenarios better than traditional approaches, paving the way for their adoption in clinical settings. Importantly, the incorporation of machine learning into clinical decision-support systems could also enhance discussions with patients and families about prognosis and treatment options, thus actively involving them in care decisions. Overall, our study contributes to the precision medicine approach, shifting the paradigm from a one-size-fits-all model to a more tailored, data-driven stroke treatment strategy.

The significance of feature importance as determined by our lasso regression model holds substantial implications for understanding and managing ischemic stroke. Notably, the “Admission NIHSS Score” emerged as a potent predictor, affirming the score’s established role as a critical index of stroke severity and its influence on outcomes (19, 20). This highlights the necessity for immediate and precise assessment at the time of admission, directing resources and attention to those most at risk.

The negative impact of “Onset To Needle Time” on patient outcomes further underscores the “time is brain” doctrine in stroke care, emphasizing the importance of swift treatment (21, 22). Our findings reinforce existing protocols advocating for rapid response and treatment initiation, which are vital in minimizing neuronal loss and improving functional recovery.

Additionally, the model illuminated the lesser but still significant roles of factors such as “Age” (23) and “Swallowing Function Score” (24), suggesting a multifactorial approach is needed in patient evaluation. Understanding these variable influences could aid in developing comprehensive care plans that address both medical and rehabilitative needs.

This nuanced appreciation of feature importance not only guides clinicians in identifying high-risk patients but also serves to inform the development of future stroke care guidelines and policies. It propels a move towards an integrated care pathway where multiple factors are considered in concert to optimize patient outcomes.

The integration of machine learning models into clinical practice holds promise for enhancing post-thrombolysis care for ischemic stroke patients. The precise risk stratification provided by our lasso regression and MLP models can help clinicians tailor treatment plans, potentially improving patient outcomes. In practical settings, this might streamline the decision-making process for interventions and enable more efficient use of healthcare resources. However, the real-world application of these models necessitates careful consideration regarding their implementation into clinical workflows, the training of medical staff, and the ongoing evaluation of their impact on patient care.

The study’s limitations are an integral part of its overall assessment and future research directives. Primarily, the retrospective nature of the study and the use of data from a single region may limit the generalizability of the findings to other populations. The potential for selection bias and overfitting of models cannot be overlooked and may affect the predictive accuracy when applied to a broader cohort. Moreover, our analysis may have overlooked certain influential variables, including wider sociodemographic factors and detailed post-admission treatment protocols, which could significantly affect patient outcomes.

As for future research, prospective studies are warranted to validate the predictive models in diverse populations and across multiple healthcare settings. Future studies should also aim to include a broader range of variables, including genetic markers, lifestyle factors, and long-term patient follow-up, to understand the sustained impact on stroke outcomes. The exploration of machine learning interpretability and the development of dynamic models that evolve with new data inputs will be critical in enhancing the practical utility of these predictive tools. Furthermore, research should investigate the integration of predictive models into clinical decision support systems and evaluate their actual impact on clinical decision-making and patient outcomes.

## Conclusion

In conclusion, our study underscores the potential of machine learning models, particularly lasso regression and MLP, to enhance predictive accuracy for END following thrombolysis in ischemic stroke patients. These findings advocate for the integration of advanced analytics into clinical practice, promising a shift towards more personalized and effective stroke management. Future endeavors should focus on addressing the limitations identified, broadening the scope of research to ensure the models’ applicability and impact in diverse clinical settings.

## Data Availability

The raw data supporting the conclusions of this article will be made available by the authors, without undue reservation.
