# Supplementary material for: Machine learning-based prediction of early neurological deterioration after intravenous thrombolysis for stroke: insights from a large multicenter study
Source: Front Neurol. 2024 Sep 9;15:1408457. doi: 10.3389/fneur.2024.1408457 (PMC11416991; doi:10.3389/fneur.2024.1408457)
Supplement: Supplementary file 1 [file Table_1.DOCX]

**Supplementary Material**

1. Supplementary Fig. S1 Data Imputation Workflow for Stroke Study – *Page 2-3.*

2. Supplementary Fig. S2 Comparative Analysis of Original and Imputed Data Distributions in Stroke Study – *Page 6.*

3. Supplementary Fig. S3 Forest Plot Comparing Odds Ratios Between Original and Imputed Data for Key Variables – *Page 7.*

1. Supplementary Table S1. Comparison of Original and Imputed Data Characteristics in Stroke Study – *Pages 4-5.*


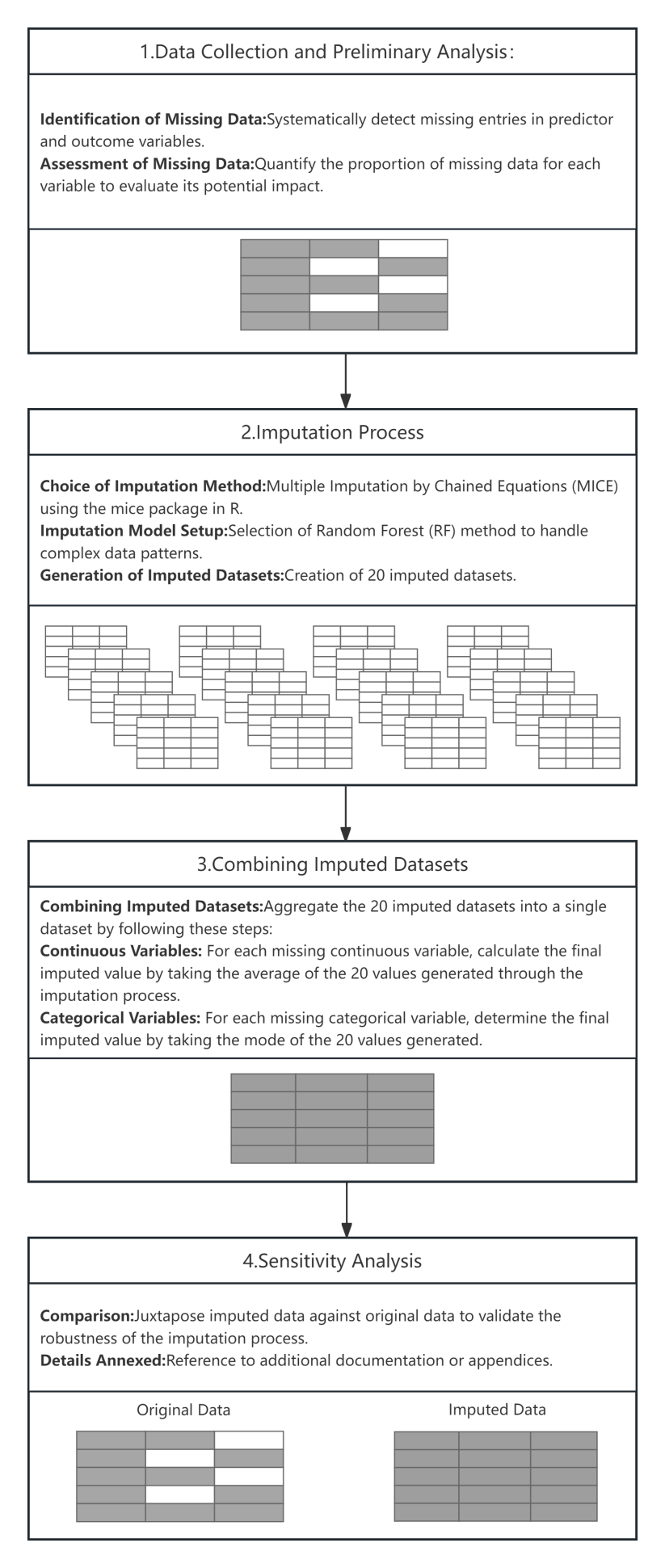


Supplementary Fig. S1 Data Imputation Workflow for Stroke Study

This figure illustrates the comprehensive data imputation workflow employed in our study on early neurological deterioration following thrombolysis for stroke. It details the process from data collection and preliminary analysis, where we identify and assess missing data in predictor and outcome variables, through the imputation phase utilizing Multiple Imputation by Chained Equations (MICE) with Random Forest to handle complex data patterns and generate 20 imputed datasets. The workflow then describes how these datasets are combined, detailing methods for aggregating imputed values for continuous and categorical variables to ensure robust data integrity, followed by a sensitivity analysis comparing imputed data against original datasets to assess the robustness and validity of the imputation process. This ensures the high quality and reliability of the data used for developing predictive models in our study.

Supplementary Table S1 Comparison of Original and Imputed Data Characteristics in Stroke Study

| Characteristic | Original Data | Imputed Data | P Value |
| --- | --- | --- | --- |
| Gender |  |  | >0.9 |
| 0 | 5,062 (70%) | 5,065 (70%) |  |
| 1 | 2,155 (30%) | 2,155 (30%) |  |
| Unknown | 3 | 0 |  |
| Age | 65 (57, 72) | 65 (57, 72) | >0.9 |
| Unknown | 3 | 0 |  |
| BMI | 24.2 (21.9, 26.2) | 24.2 (22.0, 26.1) | >0.9 |
| Unknown | 527 | 0 |  |
| Admission_mRS_Score |  |  | <0.001 |
| 0 | 3,271 (53%) | 4,121 (57%) |  |
| 1 | 845 (14%) | 909 (13%) |  |
| 2 | 487 (7.9%) | 498 (6.9%) |  |
| 3 | 416 (6.7%) | 428 (5.9%) |  |
| 4 | 925 (15%) | 1,032 (14%) |  |
| 5 | 216 (3.5%) | 217 (3.0%) |  |
| 6 | 15 (0.2%) | 15 (0.2%) |  |
| Unknown | 1,045 | 0 |  |
| Swallowing_Function_Score |  |  | <0.001 |
| 1 | 2,286 (45%) | 3,703 (51%) |  |
| 2 | 1,706 (34%) | 2,244 (31%) |  |
| 3 | 387 (7.7%) | 419 (5.8%) |  |
| 4 | 351 (7.0%) | 412 (5.7%) |  |
| 5 | 314 (6.2%) | 442 (6.1%) |  |
| Unknown | 2,176 | 0 |  |
| Onset_To_Needle_Time | 170 (125, 225) | 170 (125, 225) | 0.8 |
| Unknown | 49 | 0 |  |
| Antiplatelet_Therapy |  |  | 0.5 |
| 0 | 797 (11%) | 798 (11%) |  |
| 1 | 6,177 (89%) | 6,422 (89%) |  |
| Unknown | 246 | 0 |  |
| Anticoagulation_Therapy |  |  | 0.5 |
| 0 | 6,506 (94%) | 6,832 (95%) |  |
| 1 | 388 (5.6%) | 388 (5.4%) |  |
| Unknown | 326 | 0 |  |

This table presents a comparison between original and imputed data characteristics of patients involved in a study examining early neurological deterioration following thrombolysis for stroke. It includes data on gender, age, BMI, admission modified Rankin Scale (mRS) score, swallowing function score, time from onset to needle, and use of antiplatelet and anticoagulation therapies. The imputation process effectively addresses missing values, reducing them from various counts in the original dataset to zero in the imputed dataset, demonstrating the thoroughness of the imputation process. Statistical significance (P values) is provided to assess differences between original and imputed datasets, with most variables showing no significant difference (P > 0.9), indicating the reliability of the imputation method.

Notably, significant changes were observed in the distribution of the admission mRS score and swallowing function score. These changes are likely due to the imputation method's tendency to smooth out the distribution of categories with initially high levels of missing data. Specifically, the imputation process may have reallocated missing values based on observed patterns in the non-missing data, leading to a more even distribution across categories. This effect underscores the importance of careful interpretation when dealing with imputed data, particularly in variables where missingness was originally high.


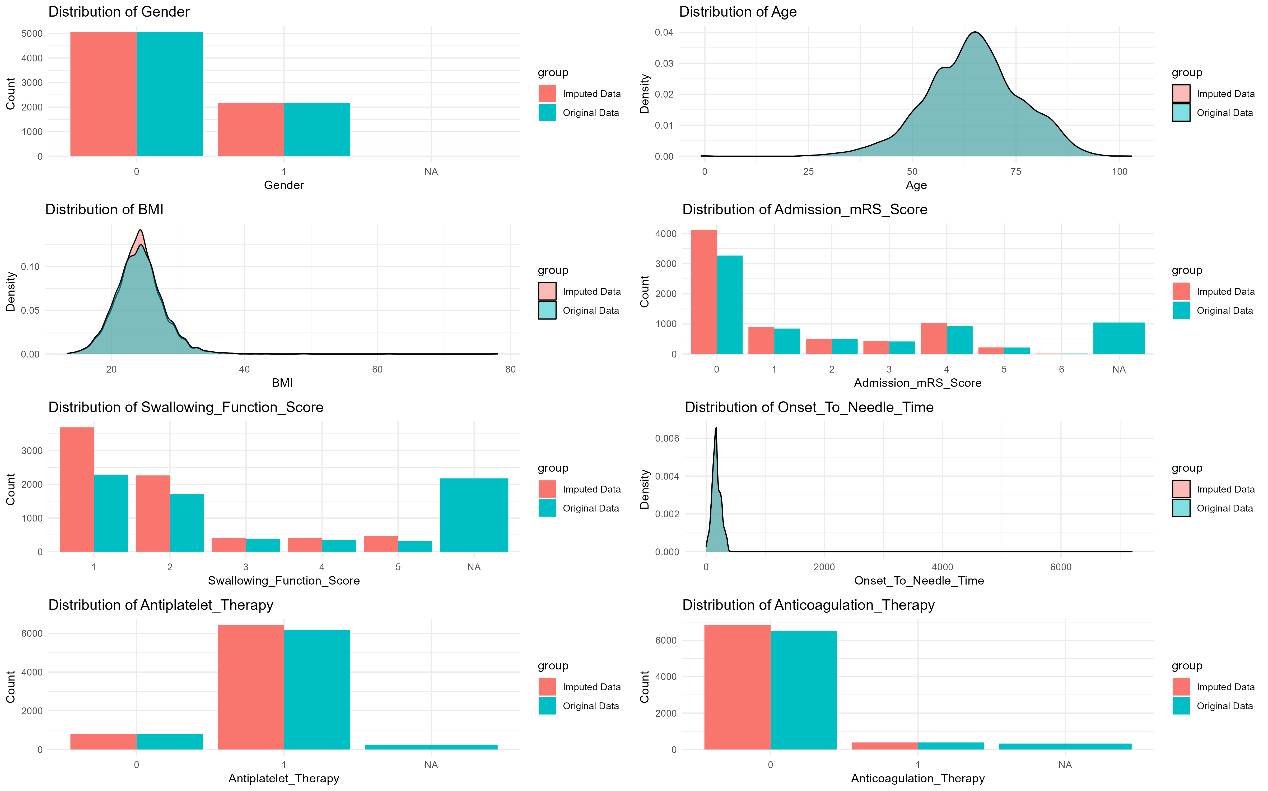


Supplementary Fig. S2 Comparative Analysis of Original and Imputed Data Distributions in Stroke Study

This figure provides a visual comparison between the original and imputed data distributions for key variables in our stroke study. The distributions are plotted for gender, body mass index (BMI), swallowing function score, age, admission modified Rankin Scale (mRS) score, time from onset to needle, and use of antiplatelet and anticoagulation therapies. The graphs highlight the effectiveness of our imputation strategy, as evident by the close alignment of the imputed data with the original data across most variables.


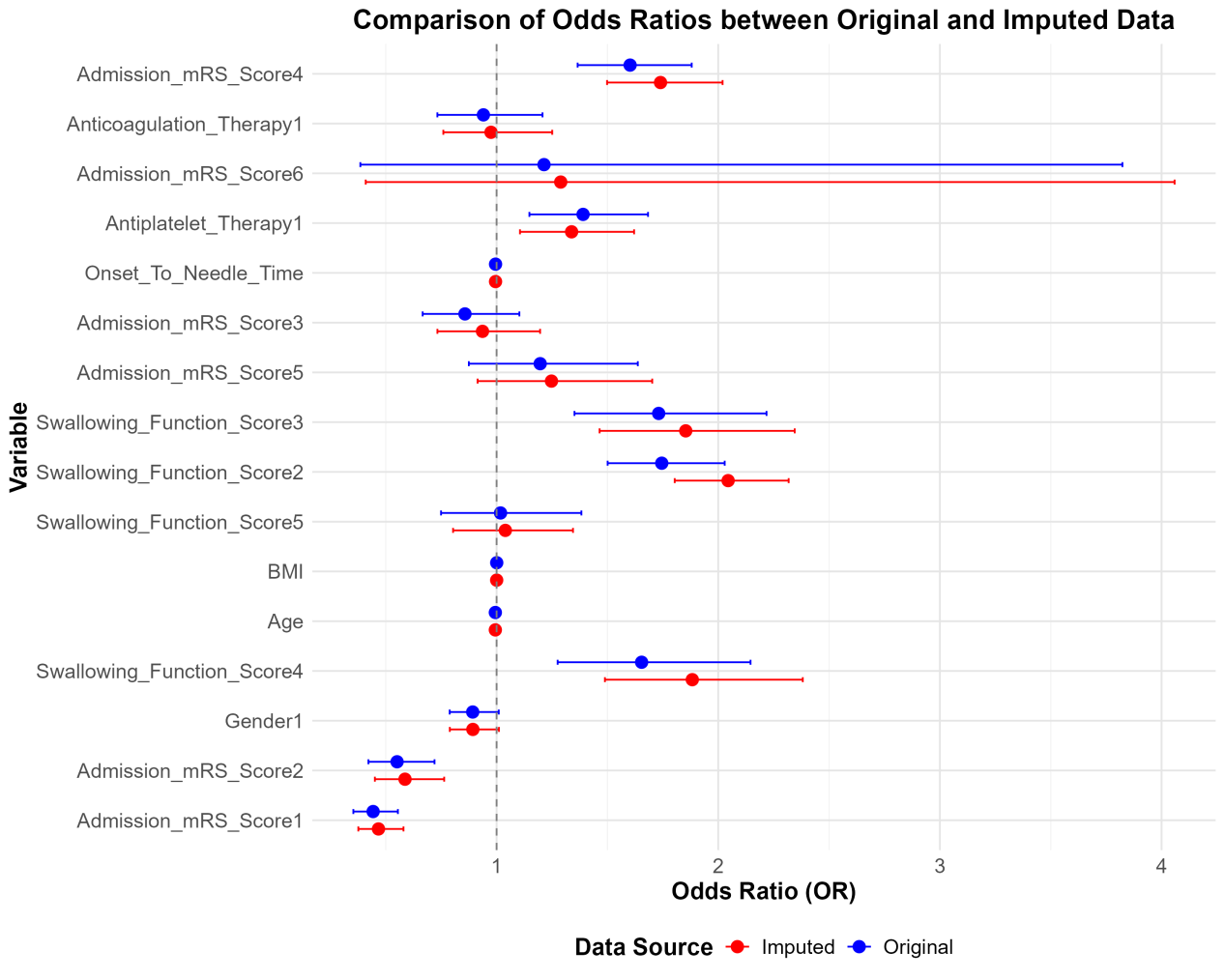


Supplementary Fig. S3 Forest Plot Comparing Odds Ratios Between Original and Imputed Data for Key Variables

This forest plot visualizes the comparison of odds ratios for key variables between the original and imputed datasets in the stroke study. The variables include admission modified Rankin Scale (mRS) scores, swallowing function scores, antiplatelet therapy, anticoagulation therapy, onset to needle time, body mass index (BMI), and age. Each variable is represented by a point estimate (odds ratio) with corresponding confidence intervals, displayed for both the original (red) and imputed (blue) data.
